# Vector Blood Meals and Chagas Disease Transmission Potential, United States

**DOI:** 10.3201/eid1804.111396

**Published:** 2012-04

**Authors:** Lori Stevens, Patricia L. Dorn, Julia Hobson, Nicholas M. de la Rua, David E. Lucero, John H. Klotz, Justin O. Schmidt, Stephen A. Klotz

**Affiliations:** University of Vermont, Burlington, Vermont, USA (L. Stevens, J. Hobson, N.M. de la Rua, D.E. Lucero);; Loyola University, New Orleans, Louisiana, USA (P. L. Dorn);; Southwestern Biological Institute, Tucson, Arizona, USA (J.O. Schmidt);; University of California, Riverside, California, USA (J.H. Klotz);; University of Arizona, Tucson (S.A. Klotz)

**Keywords:** Chagas disease, Triatominae, Triatoma, insect vectors, feeding behavior, United States, Trypanosoma cruzi, vector-borne infections, Arizona, California, *Suggested citation for this article*: Stevens L, Dorn PL, Hobson J, de la Rua NM, Lucero DE, Klotz JH, et al. Vector blood meals and Chagas disease transmission potential, United States. Emerg Infect Dis [serial on the internet]. 2012 Apr [*date cited*]. http://dx.doi.org/10.3201/eid1804.111396

## Abstract

A high proportion of triatomine insects, vectors for *Trypanosoma cruzi* trypanosomes, collected in Arizona and California and examined using a novel assay had fed on humans. Other triatomine insects were positive for *T. cruzi* parasite infection, which indicates that the potential exists for vector transmission of Chagas disease in the United States.

Chagas disease is a vector-borne disease caused by *Trypanosoma cruzi* trypanosomes. Although these parasites are rarely transmitted by insects in the United States, there is concern that vector transmission may increase (*1*). Chagas disease, endemic to most of Latin America, can be transmitted to mammals by >130 species of blood-feeding insect vectors (subfamily Triatominae). In the United States, the most common result of a triatomine bite is allergic reaction, including anaphylaxis, elicited in sensitized persons (*2*). Despite only 7 cases of vector transmission to humans reported in the United States (*3**,**4*), *T. cruzi* trypanosomes are present in >20 wildlife species. In Latin America, 8–10 million persons are infected with these parasites (*5*), and an estimated 300,000 of the ≈13 million persons from disease-endemic areas now living in the United States carry the parasite (*6*). Although vector transmission of *T. cruzi* trypanosomes is a minimal risk, 9 of the 11 triatomine species in the United States are potential vectors (*1**,**7*), and parasite transmission could increase because of climate change (*1*).

One critical aspect of transmission is parasite reservoirs; hence, the epidemiologic importance of identifying animal sources of the vectors’ blood meals, the likelihood of these vectors also feeding on humans, and their incidence of *T. cruzi* infection. Blood meals consumed by insect vectors have been detected by using several molecular techniques (*8*), but assays are challenging because of degradation of the blood in the vector’s gut, PCR inhibition, the often small size of a blood meal, and the difficulty of distinguishing multiple blood meals. We analyzed *T. cruzi* vectors collected in California and Arizona by using a novel technique— cloning following PCR amplification using universal vertebrate primers—to determine the source of blood meals and indicate the likelihood of parasite transmission to humans.

## The Study

Insects were collected by using light traps at Redington Road, Tucson, Arizona, and Escondido, California, in 2007, and within the Arizona-Sonora Desert Museum, Tucson, in 2009 (Table 1). We examined blood meals (*9*) and *T. cruzi* parasite infection (*10*) of 13 insects from 2 species of kissing bugs, *Triatoma rubida* and *T. protracta*. A mouse-fed *T. recurva* bug served as a control (*11*). Using universal vertebrate primers for *cytB* and *12S* (*12**,**13*), we identified as many blood meals as possible (Table 1). We cloned and sequenced the PCR products to isolate multiple blood meals within a single insect (Table 2). Blood meal sources were inferred by using BLAST (http://blast.ncbi.nlm.nih.gov/Blast.cgi). Pearson χ^2^ tests compared the likelihood of feeding on humans between vector species and compared the *cytB* and *12S* assays for differences in the number of blood meal taxa and blood meal haplotypes (i.e., unique DNA sequences) per insect (JMP Ver9; SAS, Cary, NC, USA).

**Table 1:** Blood meal sources of *Trypanosoma cruzi* insect vectors collected in Arizona and California, USA, 2007 and 2009, as determined by using *cytB* and *12S* rDNA assays, and haplotypes identified*

Assay and *Triatoma* spp.	*T. cruzi*	Location†	No. vertebrate blood meal sources				Haplotypes (no.) of vertebrate blood meal sources amplified in clones							No. non–blood meal clones	
			Clones	Taxa	Haplo		Human	Rat	Chick	Dog	Pig	Mouse‡		Vector§	ND
*cytB*															
* T. rubida*	–	R	8	1	1		A							6	1
* T. rubida*	–	R	10	1	2		B, C							7	1
* T. protracta*	–	E	8	1	1		D							5	2
* T. protracta*	–	E	11	1	1				A					9	1
* T. protracta*	+	E	9	0	0									7	1
* T. protracta*	+	E	8	1	2			A, B						6	
* T. protracta*	–	E	9	0	0									9	
* T. protracta*	–	E	8	0	0									7	1
* T. protracta*	+	E	8	0	0									8	
*T. recurva*‡	–		8									A (7), B			
*12S* rRNA															
* T. protracta*	+	M	8	1	2					A (7), B					
* T. protracta*	+	M	8	2	4					A (4)	A (2), B, C				
* T. rubida*	–	M	6	2	3		A (4), B			A					
* T. rubida*	–	M	7	2	2		A			A (6)					

*Vector species, *T. cruzi* infection status, collection location, number of clones sequenced, number and identity of taxa, and number of haplotypes represented in the clone sequences are indicated. Blank cells indicate clones were not found. For the *cytB* assay, the number of clones that were *Triatoma* spp. vector DNA or had uninterpretable sequences are indicated. The mouse-fed control (*cytB* assay) had 2 mouse haplotypes. Haplo, haplotypes; rat, woodrat; chick, chicken; ND, not determined because of low quality sequence data; –, negative; +, positive. †Insects were collected by using light traps at Redington Road, Tucson, Arizona (R), and Escondido, CA (E), in 2007, and within the Arizona-Sonora Desert Museum, Tucson, AZ (M), in 2009. The light traps were in “wilderness” (museum) and “sylvatic” (Redington Road and Escondido) habitats and not in human habitations. ‡Control. §*Triatoma* spp.

**Table 2:** Assays used to determine the source of blood meals and *Trypanosoma cruzi* trypanosome infection in insects collected in Arizona and California, USA*

Assay and reference	Primers, 5′ → 3′	PCR cycling†	Amplicon size
*cytB* (*12*)	cca tcc aac atc tca gca tga tga a	95°C, 40 s; 44°C, 40 s; 72°C, 40 s	358 bp
	ccc ctc aga atg att att tgt cct ca		
*12S* (*13*)	ccc aaa ctg gga tta gat acc c	95°C, 30 s; 57°C, 15 s; 72°C, 30 s	215 bp
	gtt tgc tga aga tgg cgg ta		
TCZ‡ (*11*)	cga gct ctt gcc cac acg ggt gct	94°C, 20 s; 57°C, 10 s; 72°C, 30 s	188 bp
	cct cca agc agc gga tag ttc agg		

*Insects were collected by using light traps in Tucson, Arizona, and Escondido, CA, in 2007, and within the Arizona-Sonora Desert Museum, Tucson, in 2009. For the blood meal assays, cloned PCR products (pGEM-T, Promega, Madison, WI), USA were sequenced by using the BigDye v3.1 Terminator Cycle Sequencing Kit (Applied Biosystems, Foster City, CA, USA) and analyzed by using an ABI PRISM 3730*xl* (Beckman Coulter, Fullerton, CA, USA). †For all assays: initial denaturation of 95°C for 5 m; 35 cycles of PCR and final extension of 72°C for 10 m. ‡A negative control (lacking *T. cruzi* DNA template) was included with every assay. Samples that failed to amplify were spiked with 1 μL of *T*. *cruzi* parasites boiled in 1× PCR buffer and retested to ensure that the lack of product was not caused by PCR inhibition.

Five of the 13 bugs (38%) had positive test results for human blood (Table 1); *T. rubida* bugs were significantly more likely than *T. protracta* bugs to have fed on humans (χ^2^ 9.24; p<0.01). *T. rubida* bugs had also fed on dogs and *T. protracta* bugs on woodrats (*Neotoma* spp.), chickens, dogs, and pigs. *T. cruzi* infection was found in 5/9 *T. protracta* and 0/4 *T. rubdia* bugs*.* No insect that had fed on humans was infected with *T. cruzi* trypanosomes.

The *cytB* and *12S* assays differed in the specificity of primers for vertebrate DNA and number of blood meals per insect (Figure). More than 70% of DNA cloned in the *cytB* assay was from the insect (64/87 clones); ≈10% of clones did not produce interpretable sequences. In contrast, the *12S* assay did not clone insect DNA, and all sequences were interpretable. The average number of blood meals per insect was not statistically different (*cytB* 0.56 taxa/insect, *12S* 1.75 taxa/insect; χ^2^ 8.31; p<0.10); however, the average number of haplotypes/insect was significantly higher for *12S* (*cytB* 0.78, *12S* 2.75; χ^2^ 9.09; p<0.02).

**Figure:**
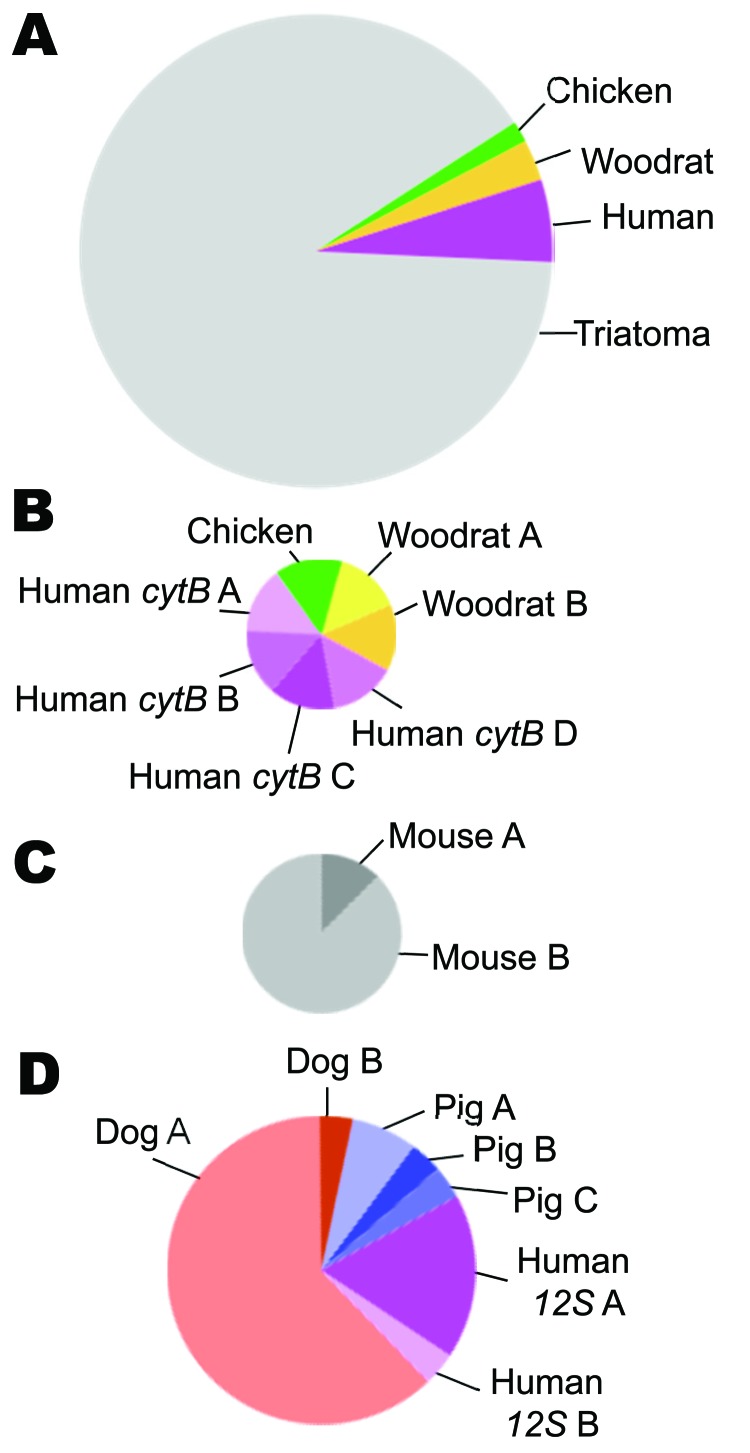
Types of blood meals found by using *cytB* and *12S* assays in insect vector species that carry *Trypanosoma cruzi*, the pathogen that causes Chagas disease, Arizona and California, USA, 2007 and 2009. Circle size is proportional to the sample size for that comparison. A) Vertebrate taxa and vector DNA (n = 71 sequences), showing that the *cytB* assay amplified vector DNA more often than blood meal DNA. B) Four vertebrate taxa among the blood meals detected by the *cytB* assay (n = 7 sequences). Unique haplotypes (DNA sequences or alleles) of human and woodrat are indicated by letters. C) Two mouse haplotypes detected in the mouse-fed control insect (n = 8 sequences). D) Types of blood meal based on the *12S* assay (n = 29 sequences).

The *cytB* assay detected more haplotypes from each blood meal taxon, indicating the bugs fed on unique individuals; 4 human and 2 woodrat sequences were all different from each other. In contrast, for *12S*, 17/18 dog sequences were identical, as were 2/3 human and 3/4 pig sequences.

The mouse-fed control (*11*) was the only insect for which no vector DNA was cloned in the *cytB* assay. All 8 clones from the control were mouse; 7 were identical. Although unexpected, heteroplasmic mitochondrial DNA has been reported for inbred mice (*14*).

## Conclusions

We found that 38% bugs of 2 species of *T. cruzi* vectors endemic to the United States, *T. rubida* and *T. protracta*, fed on humans. Infection with the Chagas parasite, *T. cruzi,* was high (55% for *T. protracta* bugs), but no insect was positive for human blood meals and the parasite. Both vectors are common in the foothills of Tucson, Arizona (*2*); although the *T. rubida* bugs in this study were uninfected, another study found that 67% of adult bugs collected around Tucson were infected with *T. cruzi* parasites (*15*).

The Arizona-Sonora Desert Museum in Tucson exhibits desert animals. All 4 insects collected from near the museum had fed on canids (dog/coyote/wolf; *Canis* spp.); 1 had fed on pigs and 2 on humans. Although canid samples are not distinguishable by *12S*, there are no dogs at the museum, so the insects probably fed on coyotes or wolves. Although javelina (*Tayassu tajacu*), a species similar to pigs, are at the museum, sequences were 99% identical to pig (*Sus scrofa*) and only a 90% match with javelina. The source of the human blood meals is not clear. No one lives at the museum, but there is camping in the area.

Around Escondido, we found *T. protracta* bugs fed on humans, woodrats, and domestic chickens, according to the *cytB* assay. This assay amplified only vector DNA from 4/7 insects, which could mean it had been a long time since the last blood meal and thus the DNA was highly degraded. We detected only 1 blood meal source in the other 3 insects from California but found 2 woodrat sequences in a single insect. Both *T. rubida* bugs collected in California had fed only on humans; 2 unique human sequences from 2 clones from 1 insect suggest it had fed on 2 humans.

Compared with *cytB,* the *12S* assay had better amplification and sequence quality and no recovery of insect vector DNA. Attempts to improve the *cytB* assay (e.g., higher annealing temperature) were unsuccessful, leading us to try the *12S* assay, which had a smaller amplicon size and higher primer specificity for vertebrate DNA (*13*). Only mouse DNA was detected from the control bug that had fed on mouse in the laboratory (*2*), demonstrating the *cytB* assay worked well for fresh blood meals; however, for degraded DNA, *12S* is a better assay.

Overall, *cytB* is more variable than *12S*, producing more haplotypes, and thus can detect feeding on multiple individuals of the same taxon. Because identifying the source of blood meals depends on the availability of similar sequences in GenBank, another advantage of *cytB* is that GenBank contains 3–4× as many vertebrate sequences for comparison.

In conclusion, although allergic reaction from triatomine bites is well known (*12*), the high incidence of human blood meals in these bugs in our study suggests that the potential for human transmission of *T. cruzi* parasites might be greater than previously thought. Our assays using vertebrate primers and cloning PCR products may be especially useful for detecting unpredicted blood meal sources and multiple blood meals.
